# A semi‐automated workflow for cohort‐wise preparation of radiotherapy data for dose‐response modeling, including autosegmentation of organs at risk

**DOI:** 10.1002/acm2.70152

**Published:** 2025-07-13

**Authors:** Louise Mövik, Anna Bäck, Kerstin Gunnarsson, Christian Jamtheim Gustafsson, Andreas Hallqvist, Niclas Pettersson

**Affiliations:** ^1^ Medical Radiation Sciences Institute of Clinical Sciences Sahlgrenska Academy University of Gothenburg Gothenburg Sweden; ^2^ Therapeutic Radiation Physics Department of Medical Physics and Biomedical Engineering Sahlgrenska University Hospital Gothenburg Sweden; ^3^ Department of Oncology Sahlgrenska University Hospital Gothenburg Sweden; ^4^ Department of Oncology Institute of Clinical Sciences Sahlgrenska Academy University of Gothenburg Gothenburg Sweden; ^5^ Radiation Physics Department of Hematology Oncology and Radiation Physics Skåne University Hospital Lund Sweden; ^6^ Department of Translational Medicine Medical Radiation Physics Lund University Malmö Sweden

**Keywords:** automation, autosegmentation, large‐scale studies, modeling

## Abstract

**Background:**

Preparing retrospective dose data for risk modeling using large study cohorts can be time consuming as it often requires patient‐wise manual interventions. This is especially the case when considering organs at risk (OARs) not systematically delineated historically. Therefore, we aimed to develop and test a semi‐automated workflow for cohort‐wise preparation of radiotherapy data from the oncology information system (OIS), including OAR autosegmentation, for risk modeling purposes.

**Methods:**

A semi‐automated workflow, including cohort‐wise data extraction from a clinical OIS, cleanup, autosegmentation, quality controls (QCs), and data injection into a research OIS was iteratively developed using 106 patient cases. We evaluated two deep learning (DL)‐based methods and compared with four atlas‐based methods for autosegmentation of the proximal bronchial tree (PBT), the heart, and the esophagus that were possible to integrate into the workflow. One method was an in‐house DL‐based model using OARs manually contoured by experts for 100 cases. Geometric and dosimetric agreements with manually contoured OARs were evaluated for 20 independent cases. The final workflow was tested on 50 independent cases.

**Results:**

The DL‐based methods were better than the atlas‐based at segmenting the PBT (mean Dice similarity coefficient (DSC) 0.81–0.83 versus 0.59–0.80) and the esophagus (mean DSC 0.76–0.77 versus 0.39–0.46). The methods performed similarly for the heart (mean DSC 0.90–0.95 (DL‐based) and 0.84–0.90 (atlas‐based)). Our in‐house autosegmentation model had the highest mean DSC for all OARs. The final version of the workflow successfully prepared data for 80% of the test cases without case‐specific manual interventions.

**Conclusions:**

The semi‐automated workflow enabled efficient cohort‐wise preparation of OIS data for risk modeling purposes. Our in‐house DL‐based segmentation model outperformed the other methods.

## 1 INTRODUCTION

The time associated with manual interventions related to preparation of data for risk modeling is an obstacle hampering large‐scale studies.^1^, ^2^ In this study, we addressed the issue of time required to retrospectively prepare radiotherapy (RT) data in the oncology information system (OIS), such as dose to delineated organs at risk (OARs), number of delivered fractions, and so forth. Henceforth, we refer to these data as OIS data. Such preparation is especially challenging when considering dose to OARs not consistently delineated historically for all patients in the study population. Autosegmentation has been both suggested and used for dose‐response modeling.^3^, ^4^, ^5^, ^6^, ^7^, ^8^, ^9^ In a large‐scale research context, unlike in the clinical setting, the objective is to exclude manual review of each case. This requires generating autosegmentations of sufficient quality. Therefore, a thorough evaluation of the study‐specific autosegmentation scenario is necessary prior to implementation.

There are several different methods for autosegmentation, mainly atlas‐based and deep learning‐based, that are available in open‐source solutions, such as CERR^10^ and 3D Slicer,^11^ as well as in commercial products.^12^, ^13^, ^14^ However, most autosegmentation methods are designed for the clinical setting and perform patient‐wise segmentation, rather than delineate OARs for an entire cohort in one session (henceforth referred to as cohort‐wise). Although patient‐wise autosegmentation is faster than manual contouring, it can still be labor intensive for a large cohort. Then, related tasks such as manually exporting each computed tomography (CT) image series from the OIS to perform autosegmentation in another software could, in total, pile up to become too time‐consuming for the study to be feasible.^1^, ^2^ In contrast to the patient‐wise strategy, a cohort‐wise approach could potentially be used to handle all the tasks that are required for preparing OIS data for modeling, from identification of study patients in the OIS to the final collection of the specific data of interest. Such cohort‐wise preparation of OIS data has the potential to alleviate the labor associated with large‐scale studies on risk modeling. To the best of our knowledge, no all‐encompassing automated solution covering all preparation tasks has been presented and tested in previous publications.

In this study, the aim was to develop and test a semi‐automated workflow for cohort‐wise preparation of OIS data for risk modeling purposes. As the workflow allows for integration of various methods for cohort‐wise segmentation of OARs, a second aim of this study was to evaluate the performance of two deep learning‐based methods for autosegmentation of the proximal bronchial tree (PBT), the heart, and the esophagus, and to compare those with four atlas‐based methods. All autosegmentation methods could potentially be integrated into the semi‐automated workflow. The considered OARs are of interest when treating patients with non‐small‐cell lung cancer (NSCLC).^15^, ^16^, ^17^

## 2 METHODS

### 2.1 Semi‐automated workflow for cohort‐wise preparation of radiotherapy data

**FIGURE 1:**
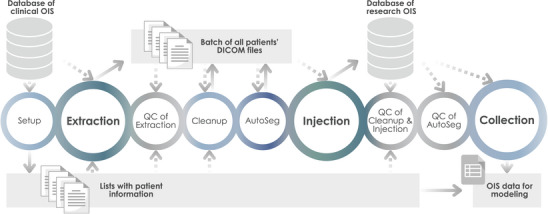
Illustration of the semi‐automated workflow for cohort‐wise preparation of radiotherapy data for risk modeling purposes. The circles represent independent automatic processes that are executed sequentially from left to right. Solid arrows indicate data generation. Dashed arrows indicate reading of data. AutoSeg, autosegmentation; DICOM, digital imaging and communications in medicine; OIS, oncology information system; QC, quality control.

We created a semi‐automated workflow for cohort‐wise OIS data preparation for risk modeling considering OARs not routinely delineated in the past (Figure 1 and Table 1). The workflow consisted of processes that together curated the data of the cohort, by data cleaning, structuring, and autosegmentation, and made it easily collectable in an OIS dedicated for research to have full ability to modify data and to avoid altering information in the clinical database. Multiple quality controls (QCs) were performed within the workflow, both integrated into processes and as separate processes. Each QC was performed at the earliest possible stage in the workflow to ensure that accurate data proceeded through the workflow. The collected data can later, together with outcome data, be used for risk modeling. The semi‐automated workflow was iteratively evaluated during development and thereafter tested using an independent test cohort (described below).

**TABLE 1:** Description of automatic processes included in the semi‐automated workflow for cohort‐wise preparation of radiotherapy data for risk modeling purposes.

Name of process	Process activities	Input data	Output data
*Setup*	Identify patients. Collect: ‐ Patient IDs. ‐ Treatment course IDs. ‐ Treatment plan IDs. ‐ Number of delivered fractions of each treatment plan. ‐ Treatment dates. ‐ Information of selected reference delineations. ‐ Create pseudonymization key.	List of considered patients or relevant search criteria, such as ICD code or phrase in ID of treatment course.	Input data to *Extraction*, *QC of Extraction, Cleanup* and *QC of Cleanup & Injection*.
*Extraction*	Pseudonymization. Extract requested data from the clinical ARIA database.	Pseudonymization key and list of considered treatment course IDs and plan IDs.	DICOM files.
*QC of Extraction*	Control that the number of files written corresponds to what was requested.	List of considered treatment plans.	Report.
*Cleanup*	Change the planned number of fractions in the treatment plans to what was delivered. Change IDs of treatment plans according to structured nomenclature. Change statuses of treatment plans and structure sets to Unapproved. Remove connections between treatment plans. Change IDs of considered OARs to the Swedish standardized nomenclature.^18^	List of treatment plans with details, such as number of delivered fractions.	Edited DICOM files.
*AutoSeg*	Segment considered OARs.	DICOM files (CT image series).	DICOM files (RT structure sets).
*Injection*	Inject files into the research ARIA database.	DICOM files.	
*QC of Cleanup & Injection*	Control that all patients have been created in the research ARIA database. Control that the number of created treatment plans is correct for each patient. Control correctness of treatment plan IDs. Control correctness of dose distributions: ‐ That all treatment plans have dose. ‐ That the delivered dose to the selected reference delineations from the treatment course are within 0.1 Gy. Control correctness of geometries: ‐ That center points for reference delineations are within 0.5 mm. ‐ That CT numbers in center points are correct.	List of considered patients and information of reference delineations.	Report.
*QC of AutoSeg*	Flag segmentation abnormalities.	IDs of considered OARs.	Report.
*Collection*	Collect RT data of interest for modeling.	List of considered patients.	Data table.

Abbreviations: CT, computed tomography; DICOM, digital imaging and communications in medicine; ICD, international classification of diseases; ID, identification; OARs, organs at risk; QC, quality control; RT, radiotherapy.

All processes in the semi‐automated workflow, except the autosegmentation, were executed as individual stand‐alone C# scripts written in Visual Studio (version 17.4.5, Microsoft, Redmond, WA, USA). Code that is not case‐specific is shared on GitHub.^19^ The scripts executed actions for an arbitrary number of patients in a single run, that is, did not need to be run separately for each patient. All processes are described next as well as in Table 1.

The process *Setup* was designed to identify patients of interest in the database of the clinical OIS (ARIA version 16.0 and 18.0, Varian Medical Systems, Palo Alto, CA, USA), either from a list of patient identifications (IDs), or using structured query language (SQL) and the Eclipse scripting application programming interface (ESAPI, Eclipse version 16.01.10 and 18.0, Varian Medical Systems, Palo Alto, CA, USA). Information of interest for each patient (listed in Table 1), for example, treatment plan IDs, number of delivered fractions, and details of a selected existing structure (henceforth referred to as the reference delineation), to be used later in the workflow were collected and stored in lists.

With one click, the *Extraction* process extracted the relevant data (treatment plans, dose distributions, CT image series, and RT structure sets) for all patients in the cohort from the clinical ARIA database and wrote it to digital imaging and communications in medicine (DICOM) files. The script utilized a DICOM daemon, which is a background service that was set up to facilitate the sending and receiving of DICOM files. Furthermore, DICOM Toolkit (DCMTK) executables were used to send data.^20^ The patients were also pseudonymized in this process.

The extraction result was checked in the *QC of Extraction* process. Here, a script was executed that controlled whether the number of written DICOM files of each modality (CT image slices, treatment plans, dose distributions, and RT structure sets) corresponded to what was requested to be extracted for each patient according to the list from the process *Setup*. Patients with disagreement between extraction request and extraction result were automatically flagged for manual review.

In *Cleanup*, the extracted DICOM files were cleaned (see actions in Table 1 and affected DICOM tags in code^19^) to strive for structured, correct, and easily accessible data to end up in the research ARIA database (version 16.0 and 18.0). For example, the status of the treatment plan was changed to unapproved to allow full ability to edit treatment plan parameters. Another example is that the planned number of fractions in each treatment plan was changed to the number of delivered fractions, and the dose distribution rescaled accordingly.

The process *AutoSeg* was designed to include a method for cohort‐wise autosegmentation of OARs. The method must be capable of performing OAR segmentation for an entire cohort in one session to be feasible to integrate into the workflow. In other words, the method must support patient‐wise automatic execution with scripting (rather than manually initiating autosegmentation individually for each patient) or cohort‐wise manual execution (autosegmentation initiated for the entire cohort with one click). Different methods for cohort‐wise segmentation of the PBT, the heart, and the esophagus with potential to be integrated into the workflow were evaluated separately (Section 2.2).

The *Injection* process was designed to, in a single run, inject all DICOM files for all patients in the cohort into the research ARIA database. The script included the operation C‐STORE from the C# library Evil‐DICOM.^21^

In *QC of Cleanup & Injection*, a script was executed that performed controls (listed in Table 1) to verify that the expected data had been correctly created in the research ARIA database. For example, the information about the reference delineation (including the mean dose, its center point, and the CT number in the center point) that had been collected in the clinical ARIA database was checked to match the values of the corresponding delineation in the research ARIA database. Patients with identified issues were automatically flagged for manual review.

The process *QC of AutoSeg* is intended for controls focused on the correctness of the generated autosegmentations. The controls must not require manual reference contours and must be possible to automatically execute. One example of an advanced QC method possible to include in this process is a separately trained QC‐model to predict DSC.^22^, ^23^ Other examples, which are possible if you use your own trained deep learning‐based model for autosegmentations, are to threshold the softmax outputs from the model inference results^24^, ^25^ or use Monte Carlo dropout during inference to estimate uncertainty.^26^, ^27^ It was beyond the scope of this study to optimize an advanced QC for detection of autosegmentation anomalies. To be able to evaluate a complete workflow in this work, we integrated a simplified QC based on the OAR volumes as an example. Our simplified QC was separately assessed for the evaluated autosegmentation methods as described in Section 2.2.3. The information from the autosegmentation to use in the QC can be retrieved from the database of the research OIS (as depicted in Figure 1) or directly from the autosegmentation output before the Injection process.

Lastly, the process *Collection* extracted curated data of interest from the research ARIA database. We collected OAR mean doses as an example. Various information, for example, other metrics derived from the dose–volume histogram (DVH) or the complete DVH, can also be collected. Code examples of data collection are available on GitHub.^19^ The collected OIS data, together with outcome data, can then be used for risk modeling employing methods like logistic regression.

The performance of the semi‐automated workflow, without autosegmentation, was iteratively evaluated with 106 patient cases during development. Cases that had been flagged in a QC were manually reviewed to determine whether the issue could be automatically resolved in the next iteration. Improvements were made iteratively during the evaluation stage, resulting in the final version of the workflow. Since the other processes operate independently of the processes *AutoSeg* and *QC of AutoSeg*, their functionality can be evaluated separately. The performances of the processes *AutoSeg* and *QC of AutoSeg* were separately assessed as described in Section 2.2. The final version of the workflow, with the best‐performing autosegmentation method integrated, was tested using 50 independent cases as described in Section 2.3. Both the 106 cases used for evaluation and the 50 cases used for testing were selected from the clinical ARIA database and from a cohort of 738 patients treated with RT for NSCLC between 2002 and 2016^28^ (ethics permits with approval numbers 110–15, 2022‐04339‐02, and 2023‐04532‐02). We selected patient cases for evaluation from the whole time interval to increase the probability of encountering data management issues related to changes over time. The 50 cases used for testing were randomly sampled from the remaining patients and had no influence on the development of the workflow. While iteratively evaluating the workflow, success for a patient case was defined as accurate preparation of all requested data excluding autosegmentations in the research OIS, without the need for any case‐specific manual intervention (i.e., a manual action on a specific case in order for that case to function properly in the workflow). To evaluate the QCs within the workflow, the prepared data were reviewed by manually inspecting all patient cases (106+50 cases) in research ARIA. The manual inspection included visually controlling various IDs, that the treatment plans had dose distributions, and that the number of delivered fractions for each treatment plan was correct.

### 2.2 Evaluation of autosegmentation methods

Two deep learning‐based methods for autosegmentation of the PBT, the heart, and the esophagus were evaluated. For comparison, four atlas‐based autosegmentation methods were also evaluated. All evaluated methods could potentially be integrated into the semi‐automated workflow (as described in Section 2.1). The organs were selected because they are considered OARs of interest for thoracic RT today but may not have been systematically delineated historically, unlike the lungs.^15^

We selected 120 patient cases diagnosed with NSCLC and treated with RT (ethics permits with approval numbers 110–15, 2022‐04339‐02, and 2023‐04532‐02). For all 120 patient cases, the considered OARs were manually contoured in Eclipse (version 16.01.10) by experienced oncologists following the guidelines of RTOG 1106.^15^ Twenty of the 120 patient cases were set aside as independent test cases to use for evaluation of all segmentation methods. The test cases were treated with standard fractionated (1.7 or 2.0 Gy per fraction) RT up to 60–70 Gy. The treatment plans had been created in Eclipse (varying versions) and calculated with various versions of the pencil beam or the analytical anisotropic algorithm. The test cases were selected to have an even distribution of sexes, lung volumes, treatment years (ranging from 2002 to 2016), and slice thickness in the planning‐CT image series (ranging from 2 to 7 mm).

#### 2.2.1 Deep learning‐based segmentation

We trained our own deep learning‐based segmentation model using 100 training cases (excluding the 20 independent test cases from the previously described patient cohort). The training cases were treated in 2002–2017 and were selected to have an even distribution of sexes. The training cases had a median slice thickness of 3 mm in the planning‐CT image series. All selected training cases had both a left and a right lung. Furthermore, they were required to have a minimum distance of 3.5 cm between the top of the lungs and the end of the planning‐CT image series to ensure that the entire esophagus was included in the image. The in‐house model was trained by adopting the open‐source nnU‐Net framework.^29^ We used five‐fold cross‐validation and the three‐dimensional resolution mode. In the nnU‐Net framework, the pipeline is automatically configured based on the properties of both the training dataset (e.g., image size) and the computer setup (e.g., graphics processing unit (GPU) memory limit). Our setup included Windows (version 11), two GPUs (NVIDIA GeForce RTX 4090 and NVIDIA GeForce RTX 4080), NVIDIA driver (version 560.81), CUDA (version 12.6), Python (version 3.11.9), PyTorch (version 2.1.1+cu121), and nnU‐Net (version 2.2.1). Inference with the trained in‐house model on the 20 test cases was performed to generate segmentations of the considered OARs.

MVision AI (version 1.2.2, Helsinki, Finland) was also used to segment the considered organs on the 20 test cases. MVision AI has a deep learning‐based segmentation method that is aligned with the guidelines of RTOG 1106.^15^

#### 2.2.2 Atlas‐based segmentation

We used 20 atlas cases selected from the cohort of 100 training cases to generate the atlas‐based autosegmentation methods. The atlas cases were treated in 2017 and were selected to have an even distribution of sexes and lung volumes. All atlas cases had a slice thickness in the planning‐CT image series of 2–3 mm. In Velocity (version 4.1, Varian Medical Systems, Palo Alto, CA, USA) and RayStation (version 9B, RaySearch Laboratories, Stockholm, Sweden), custom libraries of atlases were created with our atlas cases. Velocity and RayStation have one function each for atlas‐based segmentation, both with limited opportunities to influence the segmentation method. The OARs of the 20 test cases were segmented using their inherent functions as follows. In Velocity, rigid registrations between the test case and all atlases were performed. The best‐suited atlas was selected by Velocity based on the overlap of voxels with high CT numbers. A deformable registration, including a local deformable registration around each considered OAR, between the test case and the selected atlas case was performed. Generated structures were based on the deformed OARs of the selected atlas (no fusion required). In RayStation, deformable registrations between the test case and all atlases were performed. The resulting segmentations were fused based on an inherent algorithm including majority voting and iterative exclusion of segmentation outliers. In MICE Toolkit (version 2020.2.1 [BETA], NONPI Medical AB, Umeå, Sweden), a custom workflow for autosegmentation including deformable registrations with elastix (version 0.5)^30^, ^31^ was created. Deformable registrations were performed between all test cases and all atlas cases. For each test case and OAR, one segmentation per atlas case was generated and fused to a final unique segmentation using two different methods: majority voting as well as simultaneous truth and performance level estimation (STAPLE)^32^ with a probability threshold of 50%. Properties of the deformable registration with the considered methods are presented in Table 2.

**TABLE 2:** Properties of the deformable registrations with the considered atlas‐based methods.

Method	Transform algorithm	Interpolation method	Cost function metric	Optimizer
Velocity	B‐spline	B‐spline interpolation	Mattes Mutual Information	Regular Step Gradient Descent
RayStation	Anatomically Constrained Deformation Algorithm (ANACONDA)	Tri‐linear interpolation	Correlation coefficient	Solver developed by RaySearch
MICE Toolkit	B‐spline	B‐spline interpolation	Advanced Mean Square	Adaptive Stochastic Gradient Descent

#### 2.2.3 Evaluation of segmentation methods

The geometric agreement between the autosegmentations and the manual reference contours were evaluated with volume differences, Dice similarity coefficient (DSC),^33^ the 95th percentile Hausdorff distance (HD95)^34^, ^35^, ^36^ and the mean surface distance (MSD). The MSD represents the mean of the shortest distances from each surface point on an autosegmentation to the nearest surface point on the manual contour as well as from each surface point on the reference contour to the nearest surface point on the autosegmentation, while HD95 is the 95th percentile of those distances. Furthermore, the differences in mean absorbed doses (D_Mean_) and in near‐maximum absorbed doses (D_2%_, the minimum dose in the 2% of the volume that receives the highest dose) were evaluated. These dose parameters are commonly used variables in normal tissue complication probability (NTCP) models.^37^, ^38^ The evaluation was performed in Eclipse (version 16.01.10) and all data were extracted using scripting. Specific cases with substantial deviations were visually examined.

An example of an integrated QC for evaluating autosegmentations without relying on manual reference contours was developed and assessed. For each OAR and sex, the volume distributions of the manual reference contours from the 100 training cases were calculated and characterized by their means and standard deviations (SDs). Cases having autosegmentations with volumes falling outside the range of the mean ± 3 SD (99.7% confidence interval) for the specific OAR and sex were flagged for manual review. The QC method was evaluated by comparing the flagged cases to geometric and dosimetric metrics.

### 2.3 Test of the semi‐automated workflow including autosegmentation

The final version of the semi‐automated workflow was tested using 50 independent patient cases, as described in Section 2.1. The best‐performing autosegmentation method was integrated into the workflow when tested. Reflecting a realistic application scenario, where the workflow could be used to prepare RT data for a dose‐response study, the cases lacked manual contours of the considered OARs. Cases flagged in *QC of Extraction* or *QC of Cleanup & Injection* were manually reviewed. To confirm that no issues had been missed by *QC of Extraction* or *QC of Cleanup & Injection*, all 50 cases were visually inspected in research ARIA. Cases flagged in *QC of AutoSeg* were manually reviewed by an experienced oncologist to assess whether they represented substantial deviations. In this work, the review did not include correction of the autosegmentations. Note that autosegmentations that were not flagged in the QC were not manually reviewed. When testing the final workflow, success for a patient case was defined as neither flagged in any of the QCs nor associated with issues when visually inspected.

## 3 RESULTS

### 3.1 Semi‐automated workflow for cohort‐wise preparation of radiotherapy data

Using the first version of the semi‐automated workflow (Figure 1), we successfully injected 92% (98/106) of the patient cases used for evaluation into the research ARIA database. Five patient cases were detected lacking extracted data in *QC of Extraction*. The reason was a connection between the treatment plan and a reference point that prevented extraction from the clinical ARIA database. These reference points could not be handled automatically (functionality currently not available in ESAPI) and needed to be manually disconnected from the treatment plan before the patient cases could pass through the workflow successfully. In *QC of Cleanup & Injection*, two other patient cases were flagged. One patient case had a duplicate of the considered treatment plan created in the research ARIA database. It is not clear why this unexpected behavior during data injection occurred. Therefore, we did not revise the script for the *Extraction* process but instead handled the issue by manually deleting the duplicate (could be performed automatically in future versions). The other flagged patient case failed cleanup due to an unexpected OAR ID in the clinical treatment plan. This issue was prevented from happening again by revising the script for the *Cleanup* process in the workflow to handle that specific OAR ID. When manually inspecting the 106 created patient cases in research ARIA, we observed one patient case with an error that had not been detected in the QCs. For this case, the two treatment plans belonging to the patient had erroneously both been given the same plan ID during cleanup. The processes *Cleanup* and *QC of Cleanup & Injection* were revised to correctly handle and detect this type of issue. In summary, with the final version of the workflow, OIS data for 95% (101/106) of the patient cases used for evaluation could be successfully prepared without case‐specific manual interventions.

### 3.2 Evaluation of segmentation methods

**FIGURE 2:**
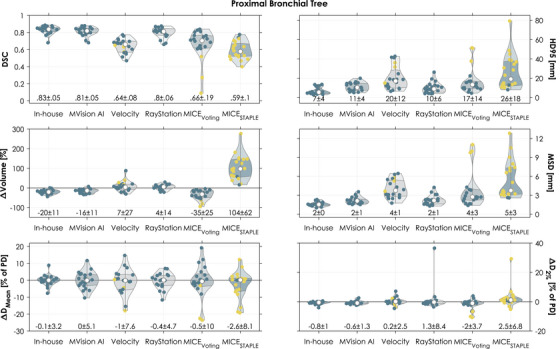
Geometric and dosimetric comparisons between each autosegmentation of the proximal bronchial tree and the corresponding manual contour (circles). Yellow circles correspond to autosegmentations flagged in the quality control implemented as an example. All doses were normalized to the prescribed dose (PD). The shape of the violin is the kernel density. In each violin, the white circle is the median and the bottom and top of the shaded area the 25th and 75th percentiles. The numbers at the bottom represent the mean ± 1 standard deviation. DSC, Dice similarity coefficient; D_Mean_, mean dose; HD95, 95th percentile of the Hausdorff distances; D_2%_, the minimum dose in the 2% of the volume that receives the highest dose; MSD, mean surface distance; PD, prescribed dose.

**FIGURE 3:**
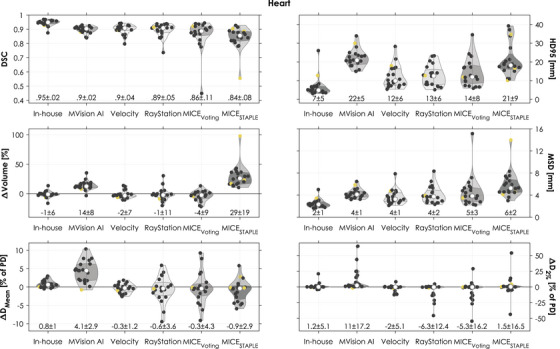
Geometric and dosimetric comparisons between each autosegmentation of the heart and the corresponding manual contour (circles). Yellow circles correspond to autosegmentations flagged in the quality control implemented as an example. All doses were normalized to the prescribed dose (PD). The shape of the violin is the kernel density. In each violin, the white circle is the median and the bottom and top of the shaded area the 25th and 75th percentiles. The numbers at the bottom represent the mean ± 1 standard deviation. DSC, Dice similarity coefficient; D_Mean_, mean dose; HD95, 95th percentile of the Hausdorff distances; D_2%_, the minimum dose in the 2% of the volume that receives the highest dose; MSD, mean surface distance; PD, prescribed dose.

**FIGURE 4:**
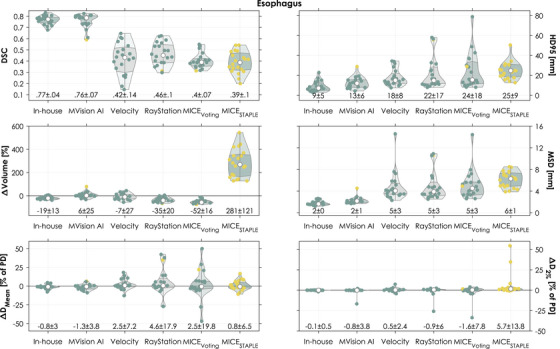
Geometric and dosimetric comparisons between each autosegmentation of the esophagus and the corresponding manual contour (circles). Yellow circles correspond to autosegmentations flagged in the quality control implemented as an example. All doses were normalized to the prescribed dose (PD). The shape of the violin is the kernel density. In each violin, the white circle is the median and the bottom and top of the shaded area the 25th and 75th percentiles. The numbers at the bottom represent the mean ± 1 standard deviation. DSC, Dice similarity coefficient; D_Mean_, mean dose; HD95, 95th percentile of the Hausdorff distances; D_2%_, the minimum dose in the 2% of the volume that receives the highest dose; MSD, mean surface distance; PD, prescribed dose.

Among the evaluated autosegmentation methods, the segmentations generated with our in‐house model were associated with best agreement for the PBT for three out of six metrics considering the mean and the SD of the differences (all metrics except for ΔVolume, ΔD_Mean_, and ΔD_2%_, Figure 2). Our model best segmented the heart according to five out of six evaluated metrics (all except ΔD_Mean_, Figure 3). Lastly, our in‐house model generated the best segmentations of the esophagus according to five out of six evaluated metrics (all except ΔVolume, Figure 4). Our in‐house model segmented all OARs with a mean MSD ≤2 mm and average dose differences ≤1.2% of the prescribed dose (PD) for both D_Mean_ and D_2%_ when compared to the manual reference contours.

The geometric agreement with the manual contours was higher for the deep learning‐based methods than for the atlas‐based methods for the PBT (Figure 2, mean DSC 0.81–0.83 vs. 0.59–0.80) and the esophagus (Figure 4, mean DSC 0.76–0.77 vs. 0.39‐0.46). The geometric agreement was similar for all methods for the heart (Figure 3, mean DSC 0.90–0.95 for deep learning‐based methods and 0.84–0.90 for atlas‐based). We observed specific cases with substantial geometric deviations, such as a HD95 of 79 mm for one PBT (MICE_STAPLE_, Figure 2) and a DSC of 0.15 for one esophagus (Velocity, Figure 4). Another noteworthy deviation was connected to a case with an unusual anatomy with increased mediastinal fatty tissue (example visualized in Figure S1). All evaluated methods except our in‐house model had difficulty with segmenting the heart for this patient case (Figure S1). For example, the heart segmentation by MICE_STAPLE_ had a volume 98% larger than the manual contour. MVision AI consistently included more of the upper regions of the heart in its segmentations (as exemplified in Figure S1). This resulted in a higher mean HD95. The volumes of the autosegmented OARs were systematically over‐ and underestimated by MICE_STAPLE_ and MICE_Voting_, respectively (Figures 2, 3, 4).

Although the geometric agreement varied among the OARs, the medians of the dose differences were both low and similar for all methods except for the heart segmented with MVision AI (≤ 0.9% of PD (deep learning‐based) and ≤ 1.5% of PD (atlas‐based) for both D_Mean_ and D_2%_ (Figures 2, 3, 4)). The heart segmented with MVision AI had a median dose difference in D_Mean_ and D_2%_ of 4.5 and 2.5% of PD, respectively. Systematic dose deviations (i.e., average dose difference for each segmentation method) ranged between −1.3% and +11% of PD (deep learning‐based) and −6.3% and +5.7% of PD (atlas‐based) (Table 3 and Figures 2, 3, 4). Random dose deviations (i.e., standard deviation of dose differences for each segmentation method) were up to 17.2% of PD for deep learning‐based methods and 19.8% of PD for atlas‐based (Table 3 and Figures 2, 3, 4). Specific cases with substantial dosimetric deviations were observed, such as a difference in D_Mean_ of 50% of PD for one esophagus (MICE_Voting_, Figure 4 and visualized in Figure S1) and a difference in D_2%_ of 64% of PD for one heart (MVision AI, Figure 3).

**TABLE 3:** Systematic (μ) and random (σ) dose deviations associated with autosegmentations generated with different methods. The deviations were based on the mean (systematic deviations) and standard deviation (random deviations) of the distributions of D_Mean_ differences and D_2%_ differences between the autosegmentations and the manual contours.

		Proximal bronchial tree		Heart		Esophagus	
		μ (% of PD)	σ (% of PD)	μ (% of PD)	σ (% of PD)	μ (% of PD)	σ (% of PD)
**ΔD_Mean_**							
	In‐house	−0.1	3.2	0.8	1.0	−0.8	3.0
	MVision AI	0.0	5.1	4.1	2.9	−1.3	3.8
						
	Velocity	−1.0	7.6	−0.4	1.3	2.5	7.2
	RayStation	−0.4	4.7	−0.6	3.6	4.6	17.9
	MICE_Voting_	−0.5	10.0	−0.4	4.3	2.5	19.8
	MICE_STAPLE_	−2.7	8.1	−0.9	2.9	0.9	6.6
**ΔD_2%_**							
	In‐house	−0.8	1.0	1.2	5.1	−0.1	0.5
	MVision AI	−0.6	1.3	11.0	17.2	−0.8	3.8
						
	Velocity	0.2	2.5	−2.0	5.2	0.5	2.4
	RayStation	1.3	8.4	−6.3	12.4	−0.9	6.1
	MICE_Voting_	−2.0	3.8	−5.3	16.2	−1.6	7.8
	MICE_STAPLE_	2.5	6.8	1.5	16.5	5.7	13.8

Abbreviations: D_Mean_, mean dose; D_2%_, the minimum dose in the 2% of the volume that receives the highest dose; PD, prescribed dose.

The evaluated QC that flagged segmented volume abnormalities (marked with yellow in Figures 2, 3, 4) detected some of the cases with substantial geometric or dosimetric deviations, but not all. Furthermore, multiple cases that were not associated with substantial geometric or dosimetric deviations were flagged.

### 3.3 Test of the semi‐automated workflow including autosegmentation

The final version of the semi‐automated workflow was tested together with our trained deep learning‐based segmentation method. The workflow successfully prepared OIS data for 80% (40/50) of the cases used for testing without requiring manual review or case‐specific manual interventions. Two patient cases had reference points that needed to be manually disconnected. Three patient cases failed extraction or injection due to settings of a specific, now obsolete, treatment machine. After modification of these settings in research ARIA, the cases successfully passed through the workflow. Five autosegmentations of the heart were flagged due to large volumes. When visually examined, four of these were determined to not represent substantial deviations. One of the flagged segmentations had an unusually large amount of fat within the pericardium. Manual inspection of the 50 cases in research ARIA that had been prepared with the final version of the workflow, confirmed that the issues detected by *QC of Extraction* and *QC of Cleanup & Injection* were valid and that no further issues had been missed. When tested, the execution time for the different processes was as follows: 4 min for *Setup*, 18 min for *Extraction*, a few seconds for *QC of Extraction*, 2 min for *Cleanup*, 49 min for *AutoSeg*, 23 min for *Injection*, 7 min for *QC of Cleanup & Injection*, a few seconds for *QC of AutoSeg* and 1 min for *Collection*. The two flagged cases with issues related to reference points took 4 min to manually handle. The three flagged cases with issues related to the settings in research ARIA took 45 min to handle. Review (without correction) of the five flagged heart segmentations took 15 min. The overall time consumption, without manual interventions, was 103 min, averaged 2 min per patient case, and resulted in prepared OIS data for 40 of 50 patient cases. Including manual interventions, the overall time consumption was 167 min, averaged 3 min per patient case, and resulted in prepared OIS data for 49 of 50 patient cases.

## 4 DISCUSSION

In this study, we developed a semi‐automated workflow for efficient cohort‐wise preparation of OIS data for risk modeling, considering OARs not systematically delineated historically. The workflow replaces manual case‐specific tasks, such as patient export from the OIS and necessary restructuring of data, and reduces manual interventions. Preparing data with the semi‐automated workflow saves time and increases the feasibility of large‐scale studies. Furthermore, we evaluated two deep learning‐based and four atlas‐based methods for segmentation of the PBT, the heart, and the esophagus that could potentially be integrated into the workflow. Our trained deep learning‐based model consistently outperformed the other methods.

The developed semi‐automated workflow was time efficient, taking on average 2 min per patient case. The data were curated by processes such as data cleaning, structuring, and autosegmentation. Additional quality‐enhancing steps, such as recalculating dose distributions using a specific dose calculation algorithm, could be integrated into the workflow. Manual inspection of the OIS data prepared with the first version of the workflow confirmed that the QCs had detected all issues except one, an issue the QCs were revised to detect. When the revised workflow was tested on 50 cases, all expected issues were found and no further issues were missed, as confirmed by the manual inspection. Errors in the data used for risk modeling reduce the accuracy of the produced model.^39^, ^40^, ^41^, ^42^ Therefore, thorough QCs capable of detecting data errors are essential. The final version of the semi‐automated workflow successfully prepared OIS data for 80% of the test cases without requiring manual review or case‐specific manual interventions. Although not all patient cases were possible to handle automatically, the ability to detect them in the QCs enabled us to perform case‐specific manual interventions so that correct data could be generated in the research OIS. In general, cases that are flagged by QCs could be directly excluded or manually reviewed. Subsequently, if reviewed cases reveal true errors, those cases could be either excluded or corrected. When integrating QCs into the data preparation process, the number of cases to flag and the approach for handling them should be optimized based on the characteristics of the specific study the data are prepared for. Such characteristics include, for example, patient cohort size and statistical power required.

The described semi‐automated workflow allows for integration of various segmentation methods. We included two deep learning‐based segmentation methods (an in‐house model and MVision AI) and four atlas‐based segmentation methods (MICE, RayStation, and Velocity). The reason for including atlas‐based methods was to compare the previously state‐of‐the‐art methods with those now considered state‐of‐the‐art, deep learning‐based methods. Our in‐house segmentation model was possible to integrate into the workflow without requiring adjustments. For the other methods, some adjustments of how the methods were used in this study would be necessary prior to integration into the workflow. For instance, in this study, each CT image series of the 20 test cases was manually exported to MVision AI, a procedure that is possible to automate with scripting. Furthermore, the autosegmentation procedures were, in this study, manually initiated in RayStation and Velocity, but the functions were either already possible to be executed with scripting (RayStation) or planned to become available with scripting in the future version (Velocity). MICE was implemented with batches of five patient cases at a time, a limit associated with our current license.

Among the evaluated segmentation methods, our trained deep learning‐based model performed autosegmentation better for all considered OARs. Specifically, for the PBT, the mean DSC and mean HD95 were 0.83 and 7 mm. These results of our segmentation model are comparable to those of another in‐house deep learning‐based segmentation model, with a reported mean DSC of 0.82 and a mean HD95 of 4 mm.^43^ To our knowledge, there have been no reports on atlas‐based segmentation results of the PBT. Autosegmentation of the esophagus is challenging due to factors such as inconsistent CT number intensities, low contrast to the surrounding tissue, and inter‐patient anatomical variability.^44^ In our study, deep learning‐based segmentation methods demonstrated substantial improvements in esophagus segmentation compared to atlas‐based methods, achieving mean DSCs of 0.76–0.77 and 0.39–0.46, respectively. The same trend was reported in the review by Liu et al.^45^ For the esophagus, they reported mean DSCs ranging from 0.71 to 0.87 and HD95 values between 4.5 and 8.7 mm.^13^, ^45^, ^46^, ^47^ Our model demonstrated comparable performance, achieving a mean DSC of 0.77 and a mean HD95 of 9 mm. For the heart, their reported values were mean DSCs of 0.87 to 0.95 and mean HD95 values of 4.6–8.0 mm.^13^, ^45^, ^46^, ^47^ Our model showed a similar performance, with a mean DSC of 0.95 and a mean HD95 of 7 mm.

In this study, the commonly used fusion algorithms majority voting and STAPLE generated segmentations that generally under‐ and overestimated the volumes of the OARs, respectively.^48^, ^49^ The results are effects of the fusion algorithms alone since the same registration result was used as a basis for each test case. The overestimation by STAPLE could be influenced by the threshold value used to generate binary images, which directly impacts the volume of the generated segmentations.^49^, ^50^, ^51^ Typically, a threshold value of 50% is used, as in this study.^32^, ^49^, ^52^, ^53^ However, contrary to our findings, Finnegan et al. reported that a 50% threshold value generally resulted in underestimated heart volumes for breast cancer patients.^49^ We also observed that MVision AI tended to include more of the upper regions of the heart in its segmentations. However, in another study evaluating MVision AI, this trend was not apparent, as indicated by a mean HD of approximately 13 mm compared to a mean HD95 of 22 mm in our study.^54^

Inaccurate autosegmentations can cause errors in the estimated doses. We have previously shown that random dose errors in the doses used for modeling result in systematic errors in the resulting NTCP model.^41^ In this study, the evaluated segmentation methods were associated with systematic dose errors ranging between −1.3% and +11% of PD (deep learning‐based) and −6.3% and +5.7% of PD (atlas‐based). Furthermore, we observed random dose errors of up to 17.2% of PD (deep learning‐based) and 19.8% of PD (atlas‐based). When using autosegmentation in a workflow for data preparation for a retrospective large‐scale study, reviewing every single autosegmentation is labor intensive and could render the study unfeasible. Therefore, the objective of an automated workflow is to minimize the need for manual reviews to enable such studies. This requires that the autosegmentation method integrated into the workflow perform well. As a complement, QCs could be used to flag specific cases suspected of substantial deviation for exclusion or manual review/correction. In this study, we showed that it was possible to integrate a QC focused on detecting segmentation anomalies into the automated workflow. The QC that we included as an example was a simplified approach based on comparison of the resulting OAR volume to expected volumes. When using autosegmentation to generate dose data for risk modeling, the QC performance for that specific study, that is, the required dose accuracy (combining both trueness and precision), should be determined. The QCs should preferably be optimized in both performance and how flagged cases should be handled, depending on how much uncertainty that can be accepted in the dose variables. Optimizing a QC is extensive work and was beyond the scope of this study. The future goal of our semi‐automated workflow is to include more advanced QCs that better distinguish autosegmentations associated with substantial deviations. Development of such QCs, using for example Monte Carlo dropout during inference for uncertainty estimation, is an active area of research.^22^, ^23^, ^24^, ^25^, ^26^, ^27^

A semi‐automated workflow for cohort‐wise preparation of OIS data for risk modeling purposes was developed. When tested together with the best‐performing autosegmentation method, a deep learning‐based model trained by us, the workflow proved to be efficient. Preparing OIS data for the tested cases took on average 2 mi per patient case, and 80% were successfully prepared without requiring manual review or case‐specific manual intervention.

## AUTHOR CONTRIBUTIONS

**Louise Mövik**: Conceptualization; data curation; formal analysis; investigation; methodology; project administration; software; validation; visualization; writing—original draft; writing—review & editing. **Anna Bäck**: Conceptualization; formal analysis; methodology; project administration; supervision; writing—review & editing. **Kerstin Gunnarsson**: Data curation; writing—review & editing. **Christian Jamtheim Gustafsson**: Methodology; software; writing—review & editing. **Andreas Hallqvist**: Conceptualization; data curation; supervision; writing—review & editing. **Niclas Pettersson**: Conceptualization; formal analysis; funding acquisition; methodology; project administration; supervision; writing—review & editing.

## CONFLICT OF INTEREST STATEMENT

This study is partly funded by a research agreement between Sahlgrenska University Hospital and Varian Medical Systems (a Siemens Healthineers company).

## Supporting information



Supporting Information

Supporting Information
