# Supplementary material for: A semi‐automated workflow for cohort‐wise preparation of radiotherapy data for dose‐response modeling, including autosegmentation of organs at risk
Source: J Appl Clin Med Phys. 2025 Jul 13;26(7):e70152. doi: 10.1002/acm2.70152 (PMC12256672; doi:10.1002/acm2.70152)
Supplement: Supplementary file 1 — Supporting Information [file ACM2-26-e70152-s002.pdf]

Example 1: Deviating esophagus and PBT

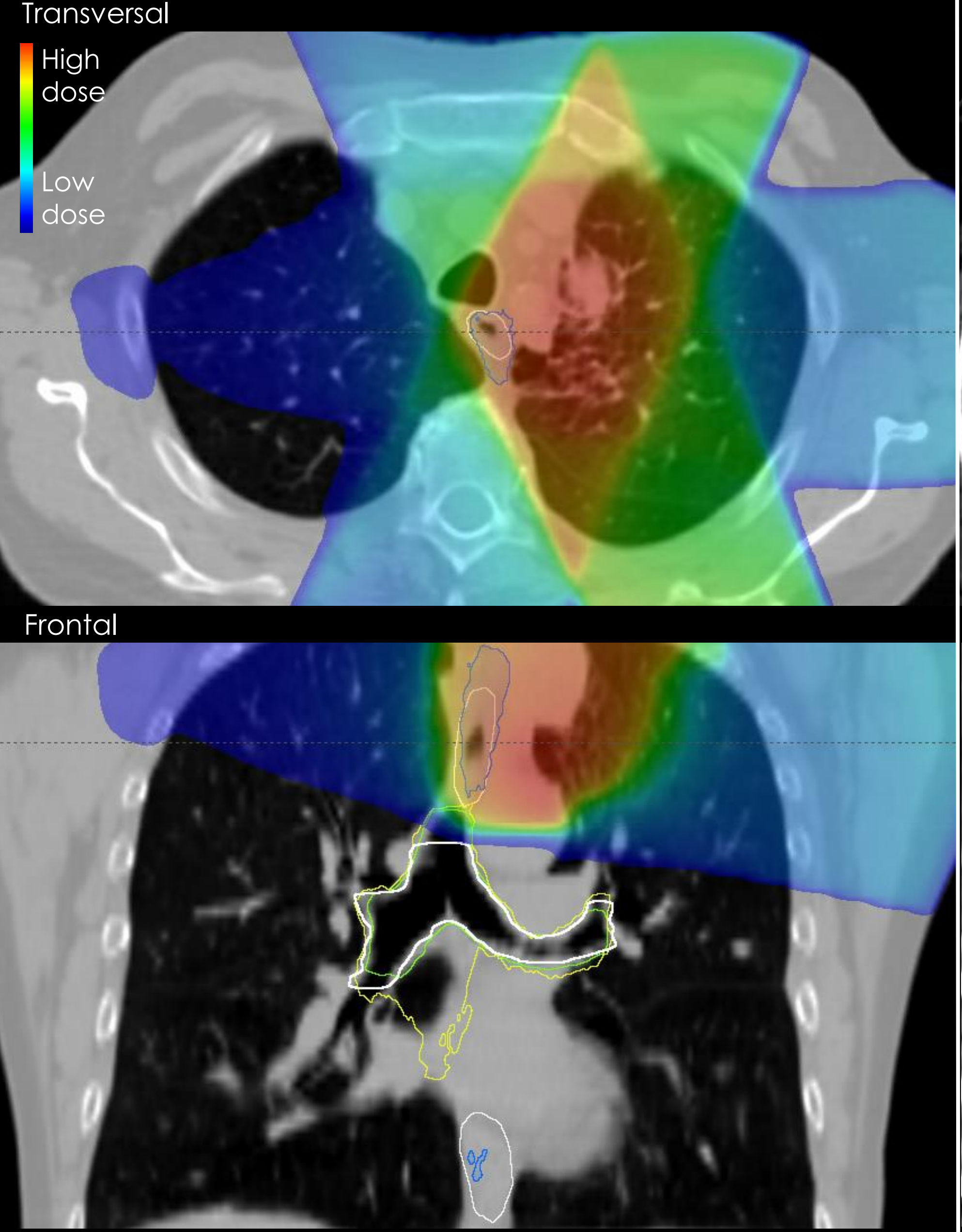

Structure	Mean dose [%]	ΔDose [% of PD]	$D_{2\%}$ [%]	ΔDose [% of PD]
**Esophagus Manual**	26.0		100.4	
**Esophagus MICE$_{Voting}$**	76.1	50.1	101.9	1.5
**PBT Manual**	3.1		7.4	
**PBT MICE$_{STAPLE}$**	4.0	0.9	36.7	29.3
**PBT RayStation**	5.9	2.8	44.0	36.6

Example 2: Deviating heart

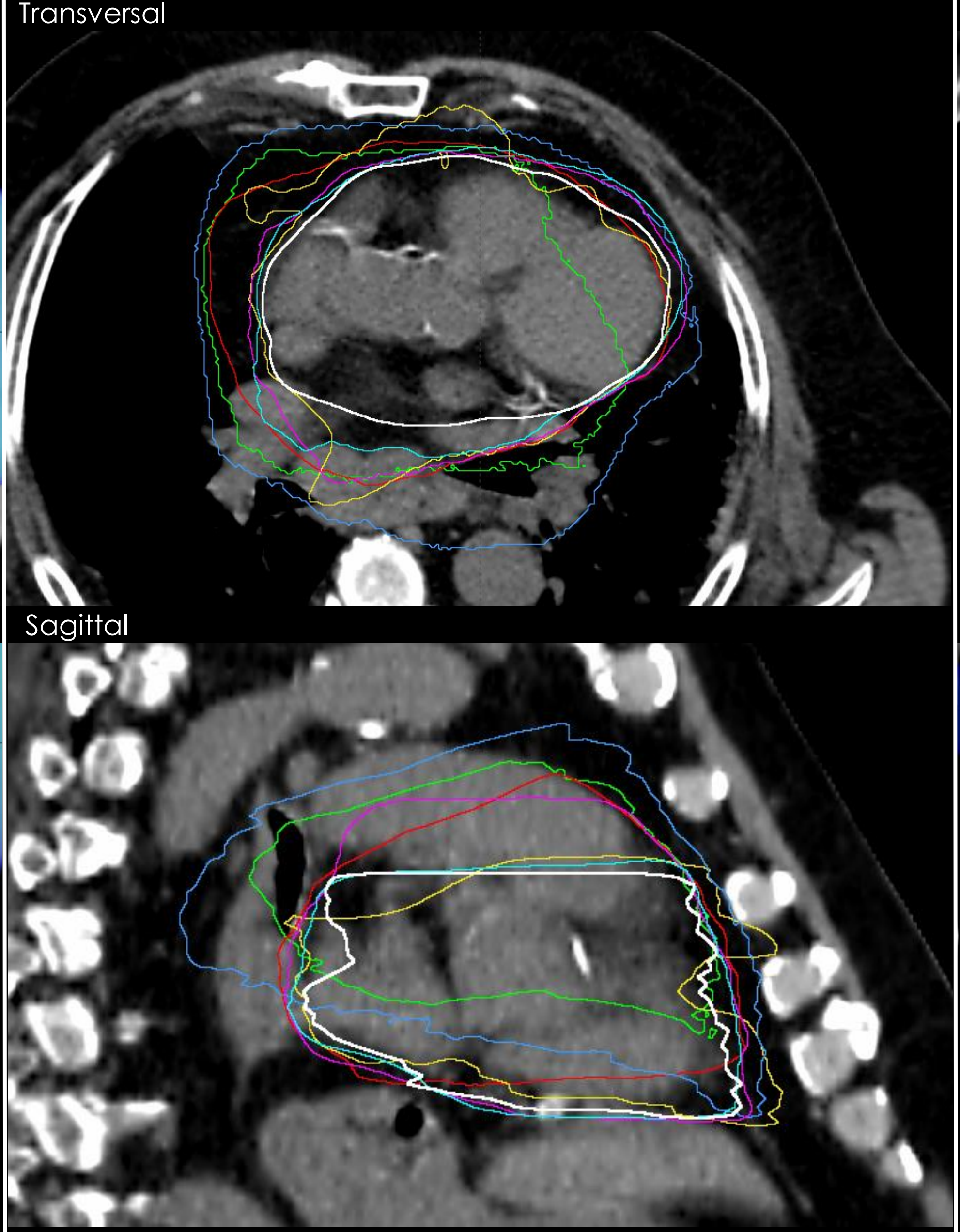

Structure	Volume [cm^3]	ΔVolume [%]	DSC	HD95 [mm]
**Heart Manual**	683.6			
**Heart MICE$_{STAPLE}$**	1353.3	98.0	0.56	34.7
**Heart MICE$_{Voting}$**	706.5	3.4	0.45	34.5
**Heart RayStation**	891.9	30.5	0.74	23.3
**Heart Velocity**	767.8	12.3	0.84	17.1
**Heart MVision AI**	925.0	35.3	0.84	19.4
**Heart Our model**	775.2	13.4	0.92	8.3

Example 3: Deviating heart

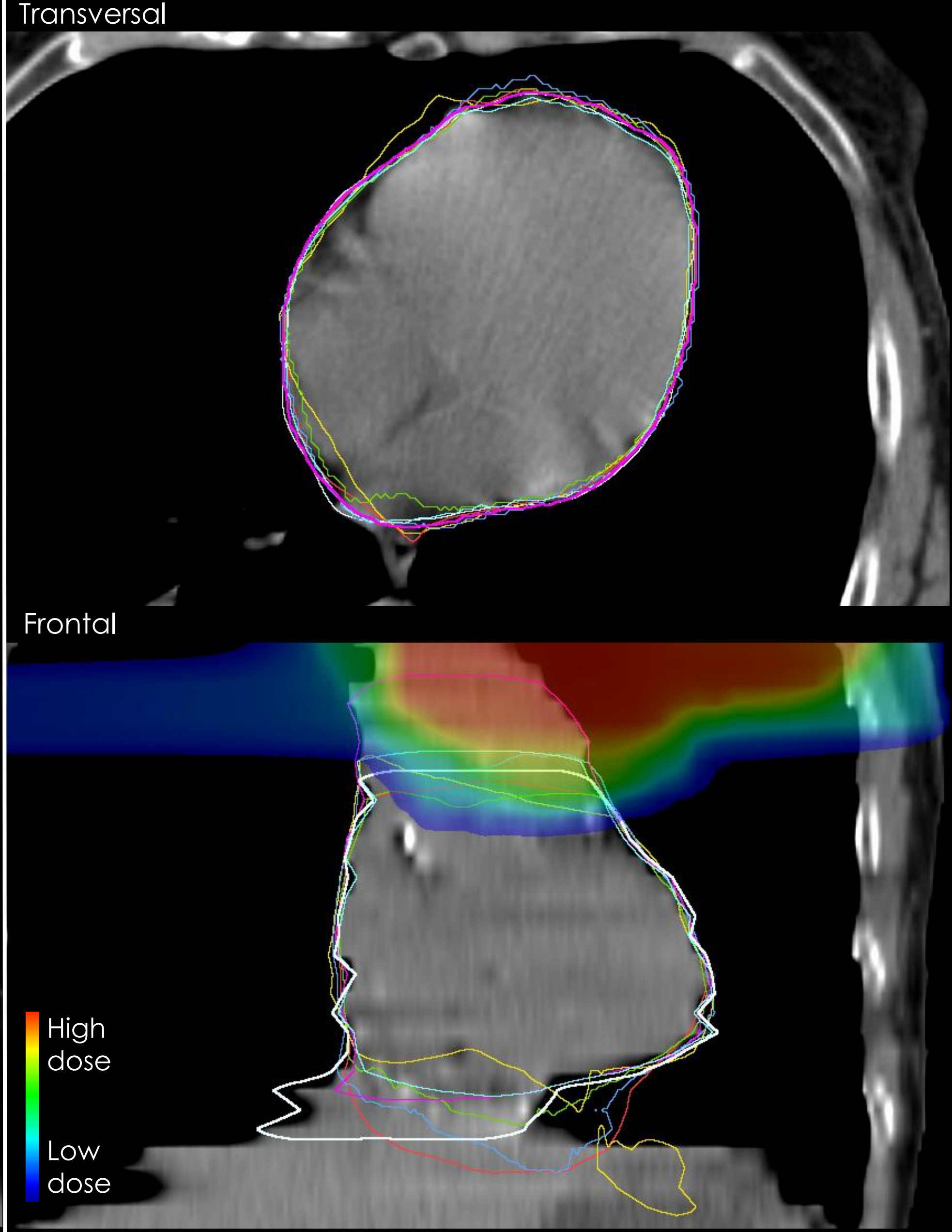

Structure	ΔDose [% of PD]	ΔDose [% of PD]	HD95 [mm]
**Heart Manual**			
**Heart MICE$_{STAPLE}$**	0.6	13.9	21.3
**Heart MICE$_{Voting}$**	-1.7	-26.7	23.0
**Heart RayStation**	-1.0	-12.5	19.4
**Heart Velocity**	-0.5	-9.3	28.3
**Heart MVision AI**	10.4	43.7	24.3
**Heart Our model**	3.0	21.0	26.1
