# Supplementary material for: A semi‐automated workflow for cohort‐wise preparation of radiotherapy data for dose‐response modeling, including autosegmentation of organs at risk
Source: J Appl Clin Med Phys. 2025 Jul 13;26(7):e70152. doi: 10.1002/acm2.70152 (PMC12256672; doi:10.1002/acm2.70152)
Supplement: Supplementary file 2 — Supporting Information [file ACM2-26-e70152-s001.docx]

**Fig S1.** Patient cases with segmentation errors resulting in dosimetric and geometric errors. *Abbreviations: D_Mean_ = Mean dose, PD = Prescribed dose; D_2%_ = The minimum dose in the 2% of the volume that receives the highest dose; DSC = Dice similarity coefficient; HD95 = 95^th^ percentile of the Hausdorff distances.*
